# SARS-CoV-2 3D database: understanding the coronavirus proteome and evaluating possible drug targets

**DOI:** 10.1093/bib/bbaa404

**Published:** 2021-01-08

**Authors:** Ali F Alsulami, Sherine E Thomas, Arian R Jamasb, Christopher A Beaudoin, Ismail Moghul, Bridget Bannerman, Liviu Copoiu, Sundeep Chaitanya Vedithi, Pedro Torres, Tom L Blundell

**Affiliations:** Department of Biochemistry, at the University of Cambridge, UK; Department of Biochemistry, University of Cambridge, Cambridge, UK; Department of Biochemistry, at the University of Cambridge, UK; Department of Biochemistry, at the University of Cambridge, UK; UCL Cancer Institute, University College London, UK; Department of Biochemistry, University of Cambridge, UK; Department of Biochemistry, at the University of Cambridge, UK; Molecular Immunity Unit, Department of Medicine University of Cambridge, MRC Laboratory of Molecular Biology, UK; Laboratório de Modelagem e Dinâmica Molecular, Instituto de Biofísica Carlos Chagas Filho, Universidade Federal do Rio de Janeiro, Rio de Janeiro, RJ, Brasil; Department of Biochemistry, University of Cambridge

**Keywords:** SARS-CoV-2 proteome modelling, SARS-CoV-2 3D database, SARS-CoV-2 drug targets, proteome analysis, drug discovery

## Abstract

The severe acute respiratory syndrome coronavirus 2 (SARS-CoV-2) is a rapidly growing infectious disease, widely spread with high mortality rates. Since the release of the SARS-CoV-2 genome sequence in March 2020, there has been an international focus on developing target-based drug discovery, which also requires knowledge of the 3D structure of the proteome. Where there are no experimentally solved structures, our group has created 3D models with coverage of 97.5% and characterized them using state-of-the-art computational approaches. Models of protomers and oligomers, together with predictions of substrate and allosteric binding sites, protein-ligand docking, SARS-CoV-2 protein interactions with human proteins, impacts of mutations, and mapped solved experimental structures are freely available for download. These are implemented in SARS CoV-2 3D, a comprehensive and user-friendly database, available at https://sars3d.com/. This provides essential information for drug discovery, both to evaluate targets and design new potential therapeutics.

## Introduction

The severe acute respiratory syndrome coronavirus 2 (SARS-CoV-2) was first detected in late 2019 in Wuhan (Hubei, China). Since then it has spread dramatically, infecting over 40 million people, with over 1 million deaths reported to date and causing major health and economic challenges globally [1]. The virus belongs to the coronavirus family that includes SARS-CoV-1 and MERS-CoV, and is characterized by a positive-sense single-stranded RNA genome. SARS-CoV-2 has approximately 79% sequence similarity to SARS-CoV-1 and 50% similarity to MERS-CoV [2]. The involvement of bats in transmission to humans is still not clear, although the sequence similarity of human SARS-CoV-2 to that of the bat coronavirus RaTG13 is ~96% [3]. High genetic variability and recombination are believed to enable widespread adaptive evolution of SARS-CoV-2 in humans around the world [4, 5].

The full genome of SARS-CoV-2 RNA was released in March 2020 (GenBank: MN908947.3) (https://www.ncbi.nlm.nih.gov/nuccore/MN908947.3)and was shown to be a single strand of 29.9 kb, with a 3′ poly (A) region and 5′-methylguanosine cap. The entry of the virus into the host cell is facilitated by the viral Spike protein, which includes a receptor-binding domain that recognizes the ACE-2 receptor on the surface of the human respiratory epithelial cell. Upon entry of the SARS-CoV-2, a single positive strand of RNA is released into the cytoplasm of the host cell [6]. The synthesis of viral RNA involves two steps: the first involves genome replication during which the RNA is transcribed via replication-transcription complexes (RTC), producing negative RNA that replicates to positive sense RNA for repackaging into the virion, and the second occurs when the negative sense RNA is transcribed via discontinuous transcription into different mRNA lengths that in turn are translated into various virion proteins. These proteins are essential for viral particle formation and for the viral replication cycle to continue [6, 7].

The SARS-CoV-2 genome can be considered as comprising three regions. The first comprises the RNA used directly as template to translate the non-structural polyproteins pp1a and pp1ab through ribosome frameshifting. The polyproteins are autoproteolytically processed into 16 non-structural proteins (Nsp1–16); these proteins also assemble to form the replication-transcription complex RTC [8]. The second region codes for the four structural proteins that occur in all coronaviruses: Spike (S), envelope (E), membrane protein (M) and nucleoprotein (N), and the third for the accessory proteins such as ORF (3a, 6, 7a, 7b, 8, 9b, 10) [9].

The SARS-CoV-2 virus, like other coronaviruses, is an obligate pathogen that uses the translation machinery of host cells for viral gene expression. It has been established that the Nsp1 viral protein of SARS-CoV-2 disrupts gene expression by inhibiting translation and promoting mRNA degradation in the host cells [10]. Recent studies [11] demonstrate interactions between Nsp1 of the SARS-CoV-2 virus and the host’s cellular proteins, such as DNA polymerase and primase subunits, thereby affecting processes such as DNA replication and damage repair. In addition, the SARS-CoV-2 viral protein Nsp2 interacts with the cap-binding protein eIF4E, an enzyme that catalyzes a key regulatory step in mRNA translation in the host. The main protease is involved in a number of cellular processes, including translation, replication, cell death and protein modification and regulation. The interactions of the viral proteins with the host’s cellular proteins have many roles including ensuring overall efficiency in the synthesis and replication of the viral proteins within the host [11]. These interactions also inhibit the host cellular mRNA processes and accelerate viral disease progression in the host [11, 12].

Various viral proteins are being studied as potential drug targets and candidates for vaccine development. Attempts to therapeutically modulate the S glycoprotein include developing antibodies against ACE2 and S protein subunits, and identifying small molecules to abolish the S-ACE2 interaction [13, 14]. The crucial role played by this interaction in viral entry requires priming of S protein by host proteases, such as the serine protease TMPRSS2, subtilisin-like furin and cathepsin L, inhibition of which is being explored [15–18]. Yet another viral structural protein of considerable therapeutic interest is the viral nucleoprotein protein that packages the viral genomic RNA. Due to its immunogenic potential, this protein is being studied as a candidate for vaccines and diagnostics [14, 19].

The viral proteases (3CL^pro^ and PL^pro^) on the other hand are Nsps, which cleave the viral polyproteins pp1a and pp1b, resulting in the formation of 16 Nsps that perform a range of important functions [20]. Nsp12–16 are believed to be involved in the crucial replication-transcription complexes of the virus along with a number of viral cofactors such as Nsps7, 8 and 10 [21, 22]. Various drug candidates that target the proteases, such as Lopinavir, Ritonavir and Disulfiram, as well as those that target the viral RNA-dependent RNA polymerase (Nsp12), including Remdesivir, Favipiravir and Ribavirin, are currently in clinical trials along with others that modulate inhibition of virus entry and control of immune response [14, 23]. The coronavirus NTPase/helicase (Nsp13), a multifunctional protein that forms part of the core replicative complex of the virus, along with Nsp12 and Nsp14 (exoribonuclease), is also considered an important target for anti-viral drug discovery. In addition to disrupting RNA secondary structures during replication, this protein is thought to catalyze the first step in 5′-cap formation of the viral mRNA [24, 25]. The subsequent steps in the process are understood to be mediated by the Nsp14 (N7-MTase domain) and Nsp16 (2′-O-MTase) [22]. In addition, a number of viral proteins have been identified to play a role in evading the host innate immune response. These proteins, including Nsps (Nsp1, Nsp9 and Nsp15), viral proteases (Nsp3 and Nsp5) and accessory proteins (ORF3b, ORF6), are thus being further investigated for the development of novel therapeutics [11, 14, 26].

Definition of the structural proteome is essential for understanding the molecular basis of any disease and for structure-guided design of new drugs. Therefore, databases act not only as a hub for experimental structures, most importantly the Protein Data Bank (PDB) [27], but also as an archive for the homology models generated by different labs, for example Genome3D [28]. Our group in the past has successfully generated bacterial structural proteome databases, for example CHOPIN for *Mycobacterium tuberculosis* [29] and Mabellini for *M. abscessus* [30], which include experimentally defined and modelled protomers and ligand interactions.

Here, we describe a novel, extensively annotated SARS-CoV-2 3D database. We focus on structures of all gene products and their higher order assemblies, i.e. homo- and hetero-oligomers, and trans-membrane regions, as well as ligand and metal-ion interactions, with acceptable assessment score. The SARS-CoV-2 3D database provides a web interface that is user-friendly and easily accessible, so that end-users can navigate, inspect and download the 3D structural proteome data, visualize modelled oligomeric complexes, analyze pockets of modelled structures and investigate SARS-CoV-2 human protein interactions, mutations and protein–ligand docking.

## Methods

### Proteome modelling

All SARS-CoV-2 modelled structures built using MODELLER [31] version (2.24). All sequences for SARS-CoV-2, obtained from GenBank: MN908947.3, were compared to structures in the Protein Data Bank (PDB) [27] using PSI-BLAST [32], which relies on Position-Specific Scoring Matrix (PSSM) profile-profile alignment, in conjunction with several multiple-sequence alignment methods, including FUGUE [33], which recognizes distant homologs using combined information from both sequence and structure, and HHsearch [34], which uses hidden Markov models (HHM). Templates for models were selected based on percentage sequence identity, amino acid sequence coverage and structure resolutions. The selected templates were re-aligned to the target sequence using Clustal Omega software [35], and the produced alignment was used in MODELLER to build the final modelled structure. All the hetero-atoms and cofactors, such as bound ligands and metal ions, were obtained from selected templates. The SARS-CoV-2 genome has nine genes annotated as trans-membrane proteins (Nsp3, Nsp4, Nsp6, ORF3a, ORF7a, ORF7b, S, E and M) and the Orientations of Proteins in Membranes (OPM) database [36] was used to annotate these transmembrane regions.

The homo-oligomeric models were built manually and automatically via the ProtCHOIR pipeline (P. Torres and T. L. Blundell, manuscript in preparation https://doi.org/10.5281/zenodo.3384945). For example, Nucleoprotein (N) has been solved experimentally in separate PDB entries; PDB ID: 6M3M is a monomer covering residues 49–174, and PDB ID: 6WZQ is a homodimer covering residues 252–364. Initially, we have built the unsolved missing regions between positions 1 and 49 using (PDB ID: 5NP3) as a template and regions between the 174 and 252 using structures from PDB IDs: 6K12, 1F15 as templates. All the regions were assembled to obtain a full protomer, followed by aligning the modelled protomer to the experimentally defined homodimer to obtain the full homodimeric modelled structure. The homodimeric S protein has been modelled in both open and the closed conformational states (Supplementary Figures S9 and S10 available online at https://academic.oup.com/bib).

Protein models of homo-oligomers such as E protein were generated using our novel modelling tool: ProtCHOIR. The ProtCHOIR pipeline relies on a homo-oligomeric-protein database and uses well-established tools such as MolProbity [37], PISA [38], GESAMT (PSI-BLAST and MODELLER to search for homologous templates, assemble the model homo-oligomer and finally assess its accuracy. This pipeline also allows for the generation of protomeric/monomeric structures to cope with the need for high-throughput comparative modelling of entire proteomes. ProtCHOIR creates oligomeric structures by performing a search on the locally created databases using PSI-BLAST before comparative modelling using MODELLER and assembling the oligomeric structure, for which a detailed report is output, documenting all the analyses performed. All generated models are assessed using MolProbity, GESAMT and PISA (in the case of oligomeric models).

The hetero-oligomer models were built based on the selected templates, where there is evidence of equivalent interactions for homologues in the literature. For example, the Nsp14-Nsp10 complex with functional ligands was built based on the experimental structures of the SARS-CoV-1 heterodimer (PDB IDs: 5C8S, 5C8T).

After obtaining the final model, side-chain energy minimization implemented in Foldit [39] was used to remove side-chain clashes. We have utilized jsPISA [40] to calculate the surface interface assemblies for only homo- and hetero-oligomeric models. The interfacial regions are characterized by parameters such as interface area (Å), solvation energy (kcal/mol), total binding energy (kcal/mol), hydrophobic log*P*-value, hydrogen bonds, salt bridges and disulphide bonds. Furthermore, the MolProbity was used as quality assessment to validate the quality of modelled structures. The MolProbity log-weighted single value is a score derived from combination of multiple features such as percentage Ramachandran not-favoured, clashscore and percentage bad side-chain rotamers.

We have not modelled proteins, such as Nsp5 and Nsp16, which have full structural coverage define by X-ray, cryo-EM and NMR experimental approaches. Structures were downloaded from the RCSB PDB, and saved as biological assemblies. We have implemented the Fpocket into our final models in order to identify potential ligands, and allosteric binding sites. The pocket ranking is based on the possibility of binding small drug-like molecules.

### Methods for predicting impacts of mutations

In order to understand the impacts of mutations on overall protein stability, where there are experimentally solved protein structures, Nsp3, Nsp5, Nsp9, Nsp12, Nsp13, Nsp15 and S, we utilized mCSM-Stability [41], mCSM-PPI [41], DeepDDG [42], PROVEAN [43], MAESTRO [44] and I-Mutant [45]. These tools either categorize the mutation as stabilizing or destabilizing or quantify the change in predicted protein folding values (ΔΔ*G*) between wild and mutant forms (ΔΔ*G* = Δ*G* wild − Δ*G*mutant). PROVEAN [43] and I-Mutant2.0 [46] are sequence-based tools, which take into account the evolutionary conservation of amino acid motifs. mCSM [41], DeepDDG [42] and MAESTRO [44] use the 3D structure of the protein to predict the thermodynamic stability changes between mutant and wild forms based on residue/atom distances, residue conservation, energy calculations and solvent accessibility. mCSM-PPI [41] was used to investigate impacts on protein–protein interactions and oligomeric interfaces. The mCSM-based tools use graph signatures to encode the atomic environment and train a predictive model, DeepDDG [42] utilizes a neural network with shared network parameters for each target residue-neighbour residue pair, and MAESTRO [44] implements a multi-agent machine learning system. The most destabilizing mutations, i.e. those reported to have the lowest average ΔΔ*G* values, are displayed in the tables corresponding to each protein on the website.

### Protein–protein docking

A list of high-confidence viral-human protein–protein interactions determined through tandem affinity purification mass spectrometry was obtained from Gordon *et al*. [11]. UNIPROT [47] identifiers for host proteins were mapped to corresponding PDB entries using the genome-scale models where available with protein structures (using the GEM-PRO pipeline implemented in SSBIO [48]), resulting in a list of 39 unique human structures for docking after quality assessment. Quality assessment involved performing pairwise sequence alignments between the UniProt sequence and the PDB sequence for each polypeptide chain in the candidate structures, candidate structures retrieved using the PDBe best structures API. Quality assessment thresholds ensured that representative structures have >90% coverage of the UniProt sequence, >95% identity to the sequence, excluding a 5% on the sequence termini and resolution <5 Å. PDB files were cleaned to remove solvents and ligands, and the highest scoring chain in the alignment selected as a representative structure. Sequence alignments and quality assessment were performed using EMBOSS Needle [49] via the SSBIO python library. Protein–protein docking for known-interaction pairs was performed using ClusPro [50], and the docked structures were minimized using CHARMM22 [51]. The top 4 docked poses with lowest CHARMM22 energy score were selected and presented into the SAR CoV-2 & Human Proteins interaction table. However, all other poses can be downloaded from the website help page.

### Ligand virtual screening

The following Nsps and their corresponding Protein Data Bank (PDB) files were screened using the FDA approved drug library of 1930 compounds extracted from the eDrug 3D web-resource [52].

(i) Nsp3 (PL Proteinase)—PDB Id 6XAA.
(ii) Nsp5 (3CLPro/Main Protease)—PDB Id 6XMK.
(iii) Nsp12 (RNA dependent RNA polymerase in complex with nsp7 and nsp8 cofactors)—PDB Id 7C2K.
(iv) Nsp14 (Guanine N7 methyltransferase). At the time of virtual screening, the crystal structure of nsp14 of SARS-CoV-2 was not solved experimentally. Hence, the crystal structure of nsp14 of SARS-CoV-1 virus with PDB Id 5C8S has been used in the virtual screening experiments as this protein has 95% amino acid sequence identity to that of SARS-CoV-2.
(v) Nsp15 (Uridylate specific endoribonuclease)—PDB Id: 6XDH.
(vi) Nsp16 (2′-O-methyltransferase)—PDB Id: 6WKQ.
(vii) S protein subunit 1 PDB Id: 6VSB.

The above-mentioned Nsps were selected for virtual screening as most of them were identified as potential drug targets in SARS-CoV-2. The proteins and the ligands were prepared using protein preparation wizard [53] and Ligprep [54] modules in Schrodinger Suite 2020-2. For 1930 drug molecules, 24 992 conformers were generated and submitted to the virtual screening workflow [54] To facilitate molecular docking with Glide [55], grid boxes were generated [56] using specific atoms of existing native/reference ligand in the active sites. Later the reference ligands were extracted from the binding sites and re-docked into the Glide-specified grid boxes to determine the differences in binding patterns between the structurally solved native pose and the docked pose. This process was repeated with each atom in the reference ligand until the lowest root mean square deviation (RMSD) was obtained between the two poses described above. Once this was achieved, the prepared drug library was docked into the active site noted by the grid with the lowest RMSD to reference. Three levels of docking were performed: high-throughput virtual screening (HTVS) was first used to select the top 10% of all the docks based on the Glide docking scores, secondly these were repeated using Glide Standard precision docking, and thirdly this was followed by the top 10% with Glide extra-precision docking. This workflow substantially reduces the number of ligand conformers to be docked by extra-precision docking, eventually reducing time and providing higher quality docking scores. The MM/GBSA [57] method was also employed to determine the ligand-binding affinities.

### SARS-CoV-2 3D web interface

The front-end of the web interface is implemented in HTML5, CSS, Bootstrap 4.5, jQuery and Font Awesome library to add icon functionality. All the tables are stored in PostgreSQL server and Express.js, a web application framework for Node.js used in the back-end to query the stored tables in PostgreSQL. We used the Embedded JavaScript template (EJS) as a template engine that dynamically generates the final HTML. The dynamic sunburst chart was created using the Plotly.js library, and the network graph interaction was created in Data Driven Documents (D3.js) package. To facilitate programmatic access to the SARS-CoV-2 3D database we have constructed a stored model, protein–protein, and PDB tables that have data available through RESTFUL APIs. The data are returned in a JSON object that is easily parsable by other software.

## Results

### SARS-CoV-2 proteome analysis

**Figure 1:**
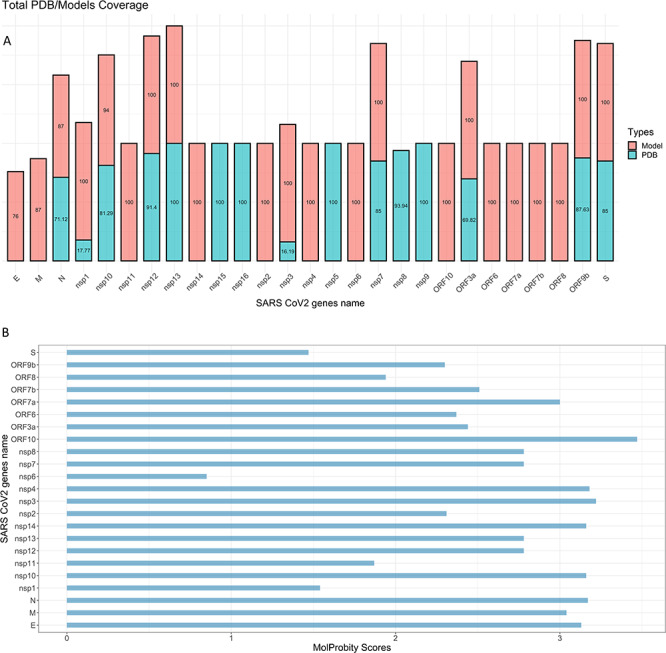
Statistical analysis of modelled proteome. (**A**) The total percentage sequence coverage of experimentally solved structures deposited in RSCB (Research Collaboratory for Structural Bioinformatics) is represented in cyan, whereas the total percentage coverage of each modelled structure is shown above it in red. (**B**) MolProbity scores for all modelled SARS-CoV-2 structures deposited in the SARS-CoV-2 3D database.

We have built oligomeric models with almost full sequence coverage for the 21 proteins that’s partially solved or do not have structures deposited in the PDB (Figure 1). The longest modelled protein is papain-like proteinase (PL^pro^) Nsp3, with 1945 residues, whereas the smallest is Nsp11 with 13 residues. The modelled structures exist as monomers, or as homo- or hetero-oligomeric complexes. The ligands and cofactors bound to the structures are retrieved from selected experimental structures of homologues. There are 15 proteins where experimentally determined structures have a mean sequence coverage is 81.41%, whereas the coverage for the modelled structures is 97.5%. The mean average of the quality assessment MolProbity score for our models is 2.52 with standard deviation of 0.65, the lowest and highest values are 0.85 and 3.47, respectively.

### Examples of SARS-CoV-2 modelled proteins

In this section, we illustrate some of the modelled structures listed in Table 1 produced using the approaches described in the Methods section. The remaining models are presented in the Supplementary Data, available online at https://academic.oup.com/bib.

**Table 1:** Structural descriptors of experimentally and modelled protein structures deposited in the SARS-CoV-2 3D database including details of coverage and quality of models and sequence coverage available from experimental methods in RCSB

Name	Model acronym	Model coverage	MolProbity score	RCSB structure available	RCSB coverage
Structural proteins					
Spike protein	S	100%	1.47	Yes	85%
Membrane protein	M	87%	3.04	No	–
Nucleocapsid protein	N	87%	3.17	Yes	71.12%
Envelope protein	E	76%	3.13	No	–
Accessory proteins					
ORF3a	ORF3a	100%	2.44	Yes	69.82%
ORF6	ORF6	100%	2.37	No	–
ORF7a	ORF7a	100%	3.00	No	–
ORF7b	ORF7b	100%	2.3	No	–
ORF8	ORF8	100%	1.94	No	–
ORF9b	ORF9b	100%	2.3	Yes	87.63%
ORF10	ORF10	100%	3.47	No	–
Nsps					
Nsp1	nsp1	100%	3.29	Yes	17.77%
Nsp2	nsp2	100%	2.31	No	–
Nsp3	nsp3	100%	3.22	Yes	16.19%
Nsp4	nsp4	100%	0.85	No	–
Nsp5	No model	–	–	Yes	100%
Nsp6	nsp6	100%	0.85	No	–
Nsp7	nsp7	100%	2.78	Yes	85%
Nsp8	nsp8	100%	2.78	Yes	93.94%
Nsp9	No model	–	–	Yes	100%
Nsp10	No model	94%	2.59	Yes	81.29%
Nsp11	nsp11	100%	1.87	No	–
Nsp12	nsp12	100%	2.78	Yes	91.40%
Nsp13	nsp13	100%	2.78	Yes	100%
Nsp14	nsp14	100%	3.16	No	–
Nsp15	No model	–	–	Yes	100%
Nsp16	No model	–	–	Yes	100%

#### Complex of Nsp14 and Nsp10

**Figure 2:**
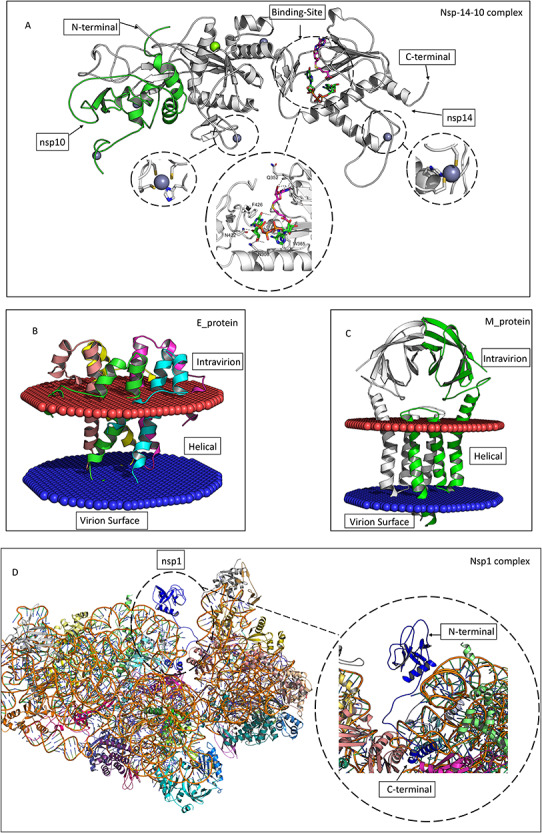
Four modelled oligomeric targets selected from the SARS-CoV-2 proteome. (**A**) Nsp14 (white-grey) and Nsp10 (green). The zinc ions are shown as silver spheres, and the magnesium ion as a green sphere. The SAH ligand is represented in magenta pink, and the G3A in green. Binding interactions of both ligands in the Nsp14 binding sites are represented as dashed lines highlighted in black. (**B**) Homo-pentameric model of Envelope protein E with each protomer indicated in a different colour. The membrane is represented as a red/blue circular structure. (**C**) Homodimeric model of the membrane protein M, with protomers coloured in green and white. The membrane is represented as a red/blue circular structure. (**D**) The structure of the Nsp1 model, coloured dark blue and encircled, complexed with the 40S ribosome (a hetero-35-mer) with the 35 proteins coloured differently.

The Nsp14, conserved throughout the CoV family, plays fundamental roles in the viral replication/transcription complex. Structurally, it has two domains, the N-terminal exoribonuclease (ExoN) and the C-terminal N7 methyltransferase domain. The Nsp14-Nsp10 complex is essential for viral replication and transcription, and disruption of the complex decreases replication fidelity [58]. The full model structure of the Nsp14-Nsp10 hetero-dimer was built based on (PDB ID: 5C8T, 5C8S) (Figure 2A). The model has a MolProbity score of 3.16, TM-score: 0.99, RMSD: 0.54 Å. Both the s-adenosyl-l-homocysteine (SAH) and guanosine-p3-adenosine-5′,5′-triphosphate (G3A) ligands, as well as the three zinc ions, are modelled from selected templates (Figure 2A). Since the selected templates are very close homologues and the TM-score between the model and the template is high, this model could serve as a reliable target in molecular docking studies as well as mutation analysis.

#### Envelope protein

The E protein, 75 amino acids long, is one of the smallest transmembrane proteins in SARS-CoV-2. The lack of E protein not only reduces viral loads but also budding of vesicles from the plasma membrane. Structurally, the E protein consists of three regions: N-terminal, transmembrane and C-terminal. The template selected for modelling the homo-pentameric E protein is PDB ID: 5X29, the NMR structure of the SARS-CoV-1 E protein [59]. The model has a MolProbity score of 3.22, TM-score of 0.99, RMSD of 0.25 Å. The transmembrane region is annotated using the Orientations of Proteins in Membranes (OPM) database (Figure 2B).

#### Membrane protein

The M protein, one of the most abundant type III glycoproteins in coronavirus particles, is located in the viral envelope between the S proteins and facilitates the virus budding [60]. Knowledge of the structure of this viral membrane protein is important for developing new therapeutics that stop the virus budding inside host cells. The M protein exists as a dimer with each protomer structurally comprising a short N-terminal region, followed by the N-domain and three transmembrane helices. It was modelled using four templates (PDB IDs: 3A7K_A, 5UTT_A, 6SPB_V, 6XDC), yielding a model with 3.04 MolProbity score. The transmembrane region was annotated using OPM (Figure 2C).

#### Nsp1

The Nsp1 plays an essential role in suppressing gene expression of the host cell via association with the ribosome. Nsp1 in SARS-CoV-1 inhibits cellular anti-viral defence mechanisms via partial shutdown of the innate immune system, so facilitating viral replication [10, 61]. Nsp1 in SARS-CoV-2 has 84% sequence identity to Nsp1 SARS-CoV-1, indicating similarity of biological function [62]. The C-terminal of Nsp1, covering residues 148-180, has recently been published as a complex with human 40S ribosomal subunit PDB ID: 6ZLW, covering residues 148–180. The modelled Nsp1 structure was generated using multiple templates PDB ID 2GDT_A, 5C5S_A, 6ZLW_i. We were able to model the full-length protein in complex with the human 40S ribosomal subunit to produce a hetero 35-mer complex. The modelled structure could plausibly serve as a target to study the effect of missense mutations on the interaction between Nsp1 and 40S ribosomal subunit (Figure 2D).

### Protein–protein docking

We modelled structures for 308 experimentally confirmed viral-human protein–protein interactions. Viral infections can, at one level, be viewed as a perturbation of host–protein interaction networks. Structural modelling of these complexes should lead to improved understanding of how SARS-CoV-2 manipulates and disrupts cellular processes. Furthermore, most antiviral development programs focus on inhibition of viral proteins [63], resulting in a small pool of targets. Inhibition of vital viral protein–human–host protein interactions provides other avenues for small-molecule development and drug repurposing screens. We have mapped the viral human protein–protein interactions using D3.js Force-Directed Graph, which provides an interactive data visualization for web browsers. The viral human protein–protein interaction data are stored in JSON format and loaded from an Application Program Interface (APIs) to produce the 2D graph. The node in each 2D graph always represented the SARS-CoV-2 protein, whereas the edges represented the human proteins. In SARS-CoV-2 3D database, the human drug target proteins are highlighted as a black arrow where human non-drug target proteins are highlighted in grey arrow.

### Small molecule ligand docking against target proteins

Top hits, scored by Glide XP [55] and MM/GBSA for receptors Nsp3, Nsp5, Nsp12, Nsp14, Nsp15, Nsp16 and S protein are included in the database as a table. Scores for all receptors were noted to be above the docking score of the reference/native ligand. Users can download the docked poses as PDB files from the web-resource and also view the docked structures on the MolStar viewer. In the viewer, docked ligands can be selected using the corresponding name/id of the ligand, located at the top of the sequence viewer. By focusing on the ligand, the user can recognize the interatomic interactions that the ligand forms with the surrounding residue environment. All possible interactions can be noted by toggling options on the ‘Representations’ menu on the right-hand panel. As noted by other groups [64, 65], we have identified Mitoxantrone, a drug that was used to treat acute myeloid leukaemia and multiple sclerosis, as a potential hit against Nsp3 in our virtual screening experiments. Protokylol, a β-adrenergic receptor agonist, was also noted among the top hits for Nsp15 and Nsp16. These screens provide initial information on the potential of repositioning of FDA-approved drugs to act on specific target proteins in SARS-CoV-2. All used anti-viral FDA drugs are mapped to DrugBank (www.drugbank.ca) with a link on the web interface.

### Mutation analysis

Single nucleotide mutations leading to amino acid changes can have a significant impact on protein structure and function. In order to understand the structural impact of mutations on the viral proteins, we used several tools, such as mCSM-Stability, mCSM-PPI, Provean, Maestro, I-Mutant and DeepDDG, to estimate the change in folding energy between mutant and native forms. We performed a saturation mutagenesis substituting each amino acid in the protein sequence on experimentally validated viral protein structures to gain a comprehensive view of the most stabilizing and destabilizing amino acid substitutions. The mutations that had, on average, the most destabilizing effect on local and global protein stability are reported. Destabilizing mutations in the vicinity of enzyme active sites or protein–ligand/protein interfaces can negatively impact necessary interactions for the viral life cycle. For example, in our analysis, several amino acid positions in different proteins were largely destabilizing regardless of the substituted amino acid. These residues were primarily located internally inside hydrophobic-rich protein domains. The most destabilizing mutated residues for S protein were found buried within the subunit 1 C-terminal and N-terminal receptor binding domains (e.g. T54, L303, G431, N439). The most destabilizing mutations for the main protease were predicted near drug binding sites (e.g. V20, V148) (supplementary S12), while the most destabilizing mutations for Nsp12 were located near cofactor binding residues (e.g. A418, K508) [66–68]. The frequently reported D614G Spike mutant was predicted to have a small destabilizing effect using mCSM (−0.210 kJ/mol) and DeepDDG (−0.129 kJ/mol), a small stabilizing effect with Maestro (0.214 kJ/mol), and a larger stabilizing effect with Provean (0.903 kJ/mol). Of note, most of the protein stability prediction tools require protein models to be in their apo forms; thus, protein stability related to glycation and cofactor binding was not evaluated in this analysis. These data can provide valuable insights into the functionality and structural robustness of different protein domains through understanding their capacity to retain stability after mutation.

### Front-end pages

**Figure 3:**
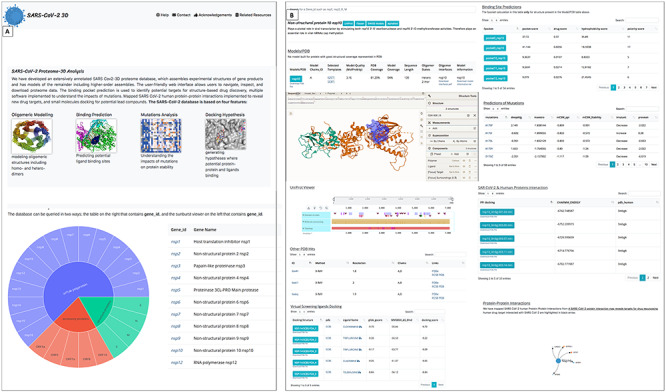
Website interface front page and result page: (**A**) Home page: the jumbotron at the top left represents the main ideas of the database. The query options such as a table or sunburst are represented below. (**B**) Results page. The top represents a brief description of the target genes with external links to other modelling pipelines: MolStar viewer for protein visualizations, Fpocket table and UniProt viewer for sequence annotation. There are five tables represented in the result pages: models, PDB structures, mutations, protein–protein interactions and virtual screening of ligands. The SARS-CoV-2 protein interaction with the human protein is annotated at the bottom of the page; the black arrows indicate human proteins annotated as drug targets.

The website can be accessed from HTTPS URL: https://sars3d.com/. The website has a ‘Navbar’ including the help page, which shows in detail how the database can be accessed programmatically through the RESTFUL API. The first page with a jumbotron identifies four features of the SARS-CoV-2 3D database: oligomeric modelling, binding-site prediction of modelled structures, mutation analysis, and protein–protein and protein-ligand docking. For simplicity, the database can be queried in two ways: through a table on the right that contains the gene identifier, or through a sunburst viewer on the left that contains the gene identifier (Figure 3A).

The results page gives a brief description of the queried gene and links to different modelling pipelines, such as SWISS Model [69], I-TASSER [70] and AlphaFold [71], to compare the different modelling approaches. The model page integrates multiple tools, such as the MolStar (Mol*) viewer that is used to view the model, PDB structures, pocket predictions and potential ligands obtained through virtual screening. The Mol* viewer shows the desired structural sequence at the top of the viewer. Calculating interactions, for example hydrogen bonding or π–π stacking, of any selected residue or ligands, can suggest interactions that will be important to consider in designing new ligands to satisfy binding-site residue interactions. The Fpocket prediction is located in a table presented under Mol* viewer; the pockets can be displayed for a target structure in the Model/PDB table in order to explore ligand binding sites. In addition, the experimentally solved structures have been annotated from RCSB and can be loaded into the Mol* viewer. A UniProt viewer [47] integrates data curated by UniProt teams, including domain and binding-site prediction, topology. This viewer has the advantage of automatically incorporating information updates from UniProt as they become available in the future.

The Models/PDB table contains a target model structure that can be viewed in the Mol* viewer and downloaded locally or programmatically through a RESTFUL API. Also, it contains other information such as templates used to build the model, MolProbity score, PDB coverage, model coverage, sequence length, oligomeric states, oligomeric interfaces and model information.

## Discussion

There are now multiple online databases specific for genomic data, including GenBank [72] containing all publicly available DNA sequences, Ensembl [73] containing annotated genomes mainly of vertebrates, and GISAID [74] containing annotated viral genomes. Most of these genome databases have manually curated data and display sequences of the target gene. In addition, there are multiple proteomic databases such as RCSB PDB for experimentally solved structures. The CATH [75] and SCOP [76] databases focus on protein domains. The UniProt database merges the sequence and structural annotations. The Catalogue of Somatic Mutation in Cancer (COSMIC) [77] is concerned with the impact of mutations in human cancer. Recently developed databases, such as Genome3D comprising collaborative work between different groups based on comparative modelling, are beginning to narrow the huge gap between 3D structures and sequence annotation.

Two 3D structural databases, developed by our group using in-house pipeline tools such as Vivace [30], are Chopin, the *M. tuberculosis* database originally established in 2015, and Mabellini for *M. abscessus* established in 2019. Since building our models, we have noted the databases such as Coronavirus3D [78], which are focused on mapping mutations to experimentally solved structures. I-TASSER, entitled Genome Wide structure and function modelling of SARS-CoV-2, provides a set of monomeric models. Swiss Model provides a set of monomeric and oligomeric models. AlphaFold provides a set of monomeric models. D3Targets-2019-nCoV a molecular docking database for SARS CoV-2 drug targets. We have provided links into our database to allow comparisons with all these major contributions.

The approach described here in the SARS-CoV-2 3D database includes not only domain or protomer structures but also multi-domain structures, homo- and hetero-oligomers, transmembrane proteins, and ligand and cofactor binding for the full SARS-CoV-2 proteome. We generated protomer structures with good sequence coverage and possible oligomeric states from selected templates. All the models in the SARS-CoV-2 3D database have good quality scores and exhibit known folds, therefore providing a reliable basis for multiple purposes, such as protein–ligand docking, protein–protein docking and mutation analysis. We have reviewed all solved experimental structures from PDB, we have mapped protein–protein interactions between SARS-CoV-2 and human proteins, considered protein–ligand docking of proved anti-viral FDA drugs, and predicted the impacts of mutations using a diverse set of tools.

Modelling the relative orientations of individual domains in multi-domain gene products remains a challenge in the field of comparative modelling. However, over the past decade, the resolution revolution in cryo-EM has proved able to provide reliable data for multi-domain and multi-component systems. Modelling the interactions of the proteins of SARS-CoV-2 with human proteins through identification of conserved amino acids located on interfacial regions is a possible solution for improving docking protein–protein interaction. We have included several methods to assess the impacts of mutations in order to indicate uncertainties and help identify false-positive predictions. We intend to update new experimentally solved structures from PDB as they become available and address all aspects of new data in future updates of SARS-CoV-2 3D.

## Conclusion

SARS-CoV-2 3D database, a comprehensive resource for the SARS-CoV-2 proteome, is based on 3D structures, using either experimentally solved structures or through comparative computational modelling based on structures of close homologues. Since the SARS-CoV-2 proteome is relatively small with 25 gene products, we have extended the methodology, used to construct our previously published databases such as Mabellini, and Chopin, to include models of transmembrane proteins, multi-domain proteins, and homo- and hetero-oligomers manually as well as using our recently developed software ProtCHOIR that is designed to build homo-oligomeric assemblies. While structural models for SARS-CoV-2 have been developed by multiple tools such as SWISS MODEL, I-TASSER and AlphaFold, our SAR-CoV-2 3D database is the first resource to our knowledge integrating not only the SARS-CoV-2 proteome model but also protein–protein interactions, mutation analysis, and protein–ligand docking, all in a user-friendly manner.

We have built an entirely new website based on Node.js with new functionality such as protein–protein interactions 2D viewer to visualize human SARS-CoV-2 interactions, UniProt viewer for sequence annotations, sunburst to navigate and query the proteome, MolStar viewer to render the 3D structure and a RESTFUL API.

The goals of the SARS-CoV-2 3D database are to bring together the most important data that help the drug discovery process by modelling unsolved proteins structures, often as oligomers, exploiting protein–protein interaction data that have recently been published. Furthermore, Fpocket is used to predict the potential ligand binding sites and potential allosteric sites. The SARS-CoV-2 3D database is regularly updated with new mutation data and a range of sequence and structure-based methods are exploited to analyze the impacts of mutations. With the likely future availability of new templates, the model structures will be updated to increase the model accuracy. Efforts are in process to add new annotations such as pathway analysis and to model further protein–protein interactions.

## Additional Information

Supplementary information is available for this paper.

**Key Points**

- SARS-CoV-2 3D is a comprehensive database for COVID-19 bringing together computational and experimental 3D structures on one platform that provides essential information for drug discovery.
- SARS-CoV-2 3D database includes protomers, homo-oligomers, hetero-oligomers, and transmembrane modelled proteins, including with ligands and cofactors.
- SARS-CoV-2 3D includes predictions of allosteric and small ligand binding sites prediction.
- SARS-CoV-2 3D includes protein–protein interactions between SARS-CoV-2 and human proteins.
- SARS-CoV-2 3D incorporates protein–ligand docking and saturation mutagenesis.

## Supplementary Material

Ali_SARS_CoV-2_supplementry_bbaa404

## Availability

All data are freely available at: https://sars3d.com/.

## Notes

**Ali F. Alsulami**, **Arian R. Jamasb**, **Chistopher Beaudoin** and **Liviu Copoiu** are PhD candidates in the Department of Biochemistry, at the University of Cambridge. Their research areas are drug discovery, computational biology and bioinformatics.

**Sherine Thomas** is a postdoc in the Department of Biochemistry, University of Cambridge, Cambridge. Her research focuses on drug discovery for infectious diseases.

**Ismail Moghul** is a PhD candidate at UCL Cancer Institute, University College London. His research areas focus on bioinformatics.

**Bridget Bannerman** is a postdoc at the Molecular Immunity Unit, Department of Medicine University of Cambridge, MRC Laboratory of Molecular Biology. Her research focuses on developing predictive models for various pathogenic micro-organisms, reviewing treatment management strategies for SARS-CoV-2 and designing tools and strategies for surveillance of antimicrobial resistance.

**Sundeep Chaitanya Vedithi** is a research director of the American Leprosy Mission and leads a group of postdoc in the Department of Biochemistry, University of Cambridge, focusing on bioinformatics and drug discovery for *Mycobacterium leprae*.

**Pedro Torres** is a professor at the Laboratório de Modelagem e Dinâmica Molecular, Instituto de Biofísica Carlos Chagas Filho, Universidade Federal do Rio de Janeiro, Rio de Janeiro, RJ, Brasil. His research focuses on bioinformatics tools for proteomic databases and virtual screening and docking for early drug discovery.

**Tom Blundell** is a professor at the Department of Biochemistry, University of Cambridge. His research focuses on structural biology, bioinformatics and drug discovery for cancer and mycobacterial infections.
