# Supplementary material for: SARS-CoV-2 3D database: understanding the coronavirus proteome and evaluating possible drug targets
Source: Brief Bioinform. 2021 Jan 8;22(2):769–80. doi: 10.1093/bib/bbaa404 (PMC7929435; doi:10.1093/bib/bbaa404)
Supplement: Ali_SARS_CoV-2_supplementry_bbaa404 [file ali_sars_cov-2_supplementry_bbaa404.pdf]

SARS-CoV-2 3D database: Understanding the Coronavirus Proteome and Evaluating Possible Drug Targets.

Ali F. Alsulami†, Sherine E. Thomas†, Arian R. Jamasb†, Christopher A. Beaudoin†, Ismail Moghul[4], Bridget Bannerman, Liviu Copoiu, Sundeep Chaitanya Vedithi†, Pedro Torres† and Tom L. Blundell

Corresponding author: Tom Blundell, Department of Biochemistry, University of Cambridge, Cambridge, CB2 1GA, UK. E-mail: tlb20@cam.ac.uk
† Contributed equally to this work.

Ali F. Alsulami, Arian R. Jamasb, Chris Beaudoin, and Liviu Copoiu are PhD candidates in the Department of Biochemistry, at the University of Cambridge. Their research areas are drug discovery, computational biology, and bioinformatics.

Sherine Thomas is a postdoc in the Department of Biochemistry, University of Cambridge, Cambridge. Her research focuses on drug discovery for infectious diseases.

Ismail Moghul is a PhD candidate at UCL Cancer Institute, University College London. His research areas focus on bioinformatics.

Bridget Bannerman is a postdoc at the Molecular Immunity Unit, Department of Medicine University of Cambridge, MRC Laboratory of Molecular Biology. Her research focuses on developing predictive models for various pathogenic micro-organisms, reviewing treatment management strategies for SARS-CoV-2 and designing tools and strategies for surveillance of antimicrobial resistance.

Sundeep Chaitanya Vedithi is Research Director of the American Leprosy Mission and leads a group of postdoc in the Department of Biochemistry, University of Cambridge, focusing on bioinformatics and drug discovery for *Mycobacterium leprae*.

Pedro Torres is a Professor at the Laboratório de Modelagem e Dinâmica Molecular, Instituto de Biofísica Carlos Chagas Filho, Universidade Federal do Rio de Janeiro, Rio de Janeiro, RJ, Brasil. His research focuses on bioinformatics tools for proteomic databases and virtual screening and docking for early drug discovery.

Tom Blundell is a Professor at the Department of Biochemistry, University of Cambridge. His research focuses on structural biology, bioinformatics and drug discovery for cancer and mycobacterial infections.

Orf1ab Polyprotein

Non-structural protein 2 (Nsp2)

Nsp2 is an RNA binding protein, involved in genome replication. The modeled structure was built using multiple templates (PDB ID: 5F22_B, 3LD1_A, 5Y81_H, 1R7G_A, 1G03_A) with a MolProbity score of 2.31.

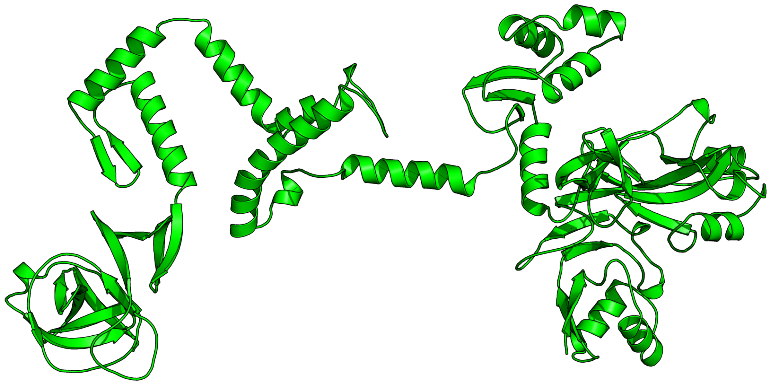

Figure S1 The Nsp2 protein structure shown in green

Papain-like proteinase (Nsp3)

Nsp3 is the largest SARS CoV-2 protein, and is a multidomain polypeptide. PL^{pro}, part of Nsp3, cleaves the N-terminus of the replicase polyprotein at a specific site to produce a functional protein. PL^{pro} is essential and considered one of the most useful drug targets[1]. The modeled structure was built using multiple templates (PDB ID: 2GRI_A, 6VXS_A, 2W2G_A, 6W9C_A, 2K87_A, 3GA8_A, 1YX1_A, 6ORH_B, 1QWG_A, 3C8F_A, 1HA8_A) with a MolProbity score of 3.22.

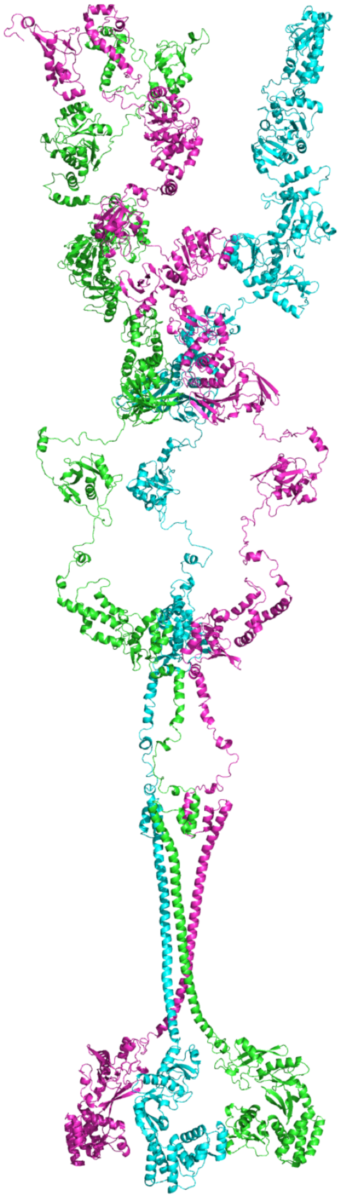

Figure S2 the assembly of multidomain homotrimer modelled Nsp3 structure.

Non-structural protein 4 (Nsp4)

Nsp4 is a transmembrane protein, vital for the host membrane rearrangement, also necessary for viral replication[2]. The modeled structure was built using multiple templates (PDB ID: 1BCP_F, 3VC8_A, 3A7K_A, 1T70_A) with a MolProbity score of 3.18.

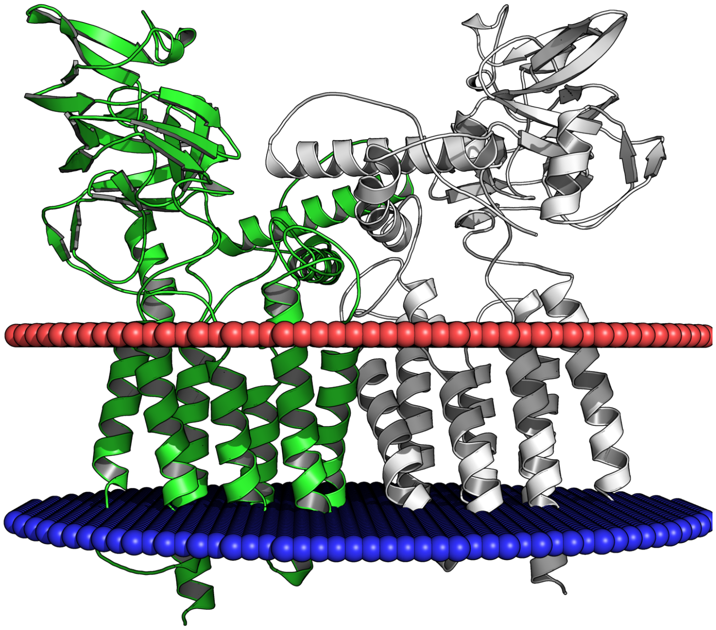

Figure S3 The Nsp4 homodimer transmembrane structure shown in green and white; the membrane is represented as a red, blue circular structure.

Proteinase 3CL-PRO Main protease (nsp5)

The main protease (M^{pro}, $3CL^{pro}$, nsp5) Cleaves the C-terminus of replicase polyprotein to functional proteins. The nsp5 very well known as a drug target. One of the best-characterized drug targets among coronaviruses is the main protease. This target has been solved experimentally, no model has been built.

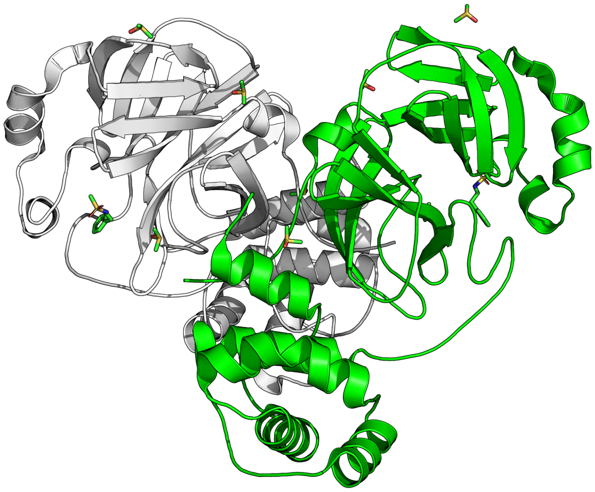

Figure S4 Experimentally solved nsp5 homodimer structure (PDB ID: 5R7Y) each chain coloured differently, the natural ligand N-(2-phenylethyl) methanesulfonamide and dimethyl sulfoxide represented in green sticks.

Non-structural protein 6 (nsp6)

It's a transmembrane protein, that has seven putative transmembrane helices. Plays important roles in host membrane rearrangement. The model structure has been downloaded from AlphaFold website[3]. To our knowledge this could be the closest 3D structure model that could describe the nsp6 protein, for this reason we have implemented into our database.

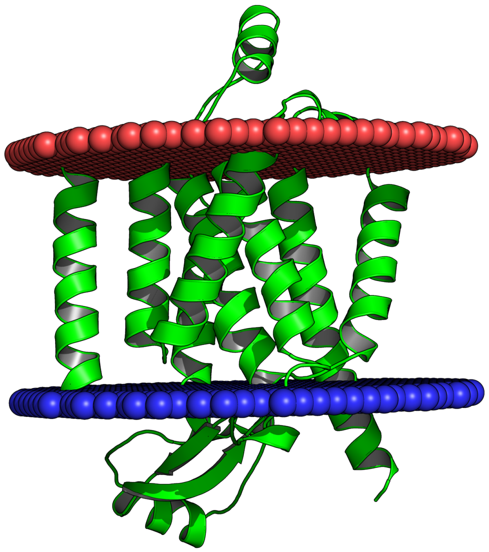

Figure S5 Alphafold prediction of nsp3 transmembrane in green the membrane is represented as a red, blue circular structure.

Non-structural protein (nsp7, nsp8, nsp12, nsp13)

The RNA-dependent RNA polymerase (RdRp) proteins are very essential for SARS CoV-2 genome replication. It consists of nsp12, nsp13, and two accessory proteins nsp7, and nsp8. This complex has been solved experimentally (PDB ID;6YYT)[4], however not with full amino acid coverage. The modeled structure built using multiple templates (PDB ID: 6M5I, 6YYT, 7C2K) with a MolProbity score of 2.78.

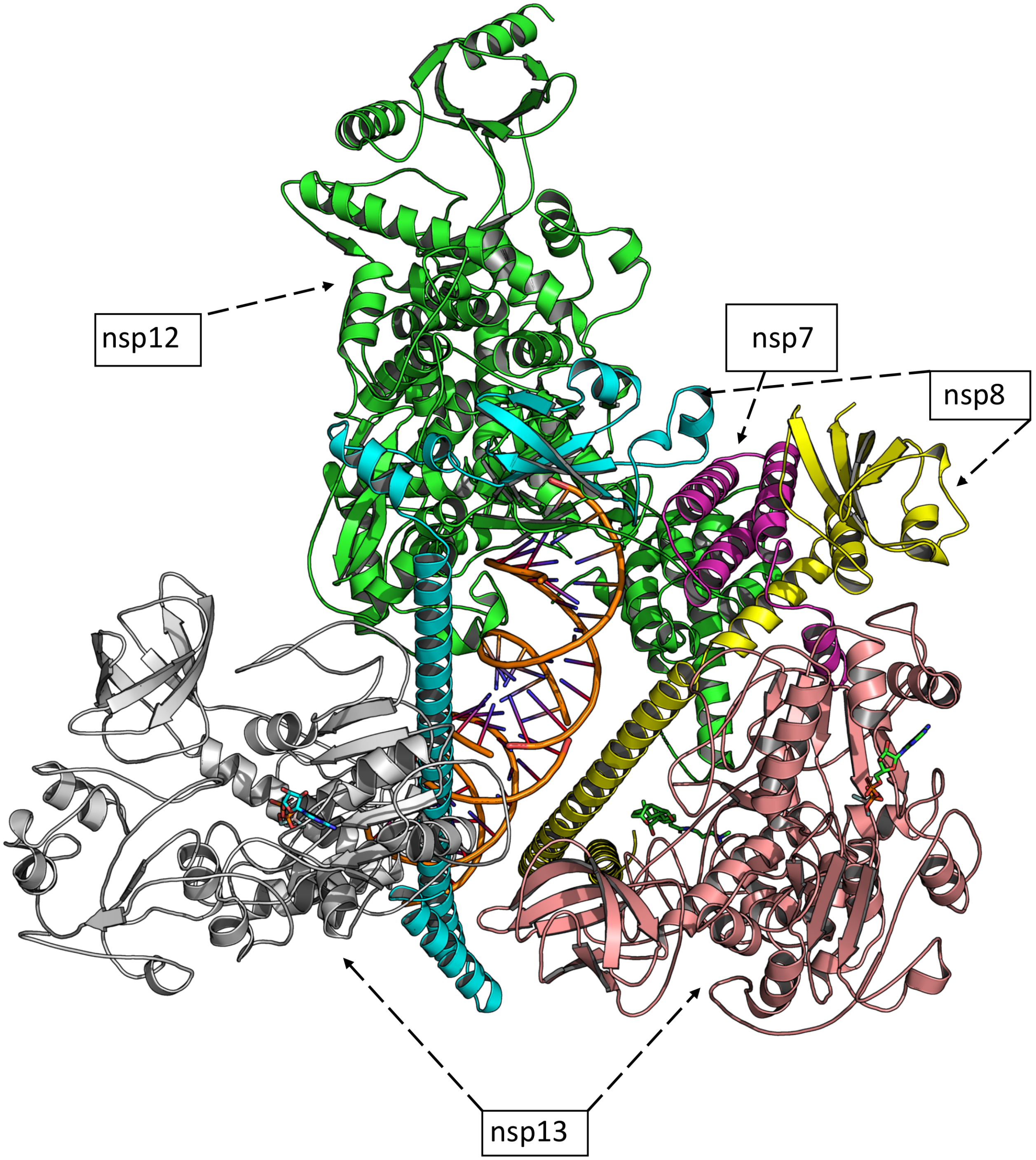

Figure S6 the RNA-polymerase is shown in green in complex with the homodimer of Nsp13 shown in white, and light red, and the homodimer of Nsp8 highlighted in yellow and cyan, whereas nsp7 shown in magenta. The ligands bound to Nsp13 are shown in stick green and cyan. The double stranded DNA, which passes through the complex, is coloured in light orange.

Non-structural protein 10 (Nsp10)

Nsp10 plays essential roles in viral transcription by interacting with other Nsp proteins, such as nsp10. The complex of Nsp10-Nsp16 has been solved experimentally (PDB ID: 6W75). In addition we have built the homo-12-mer based on (PDB ID: 2G9T) with MolProbity score of 2.59

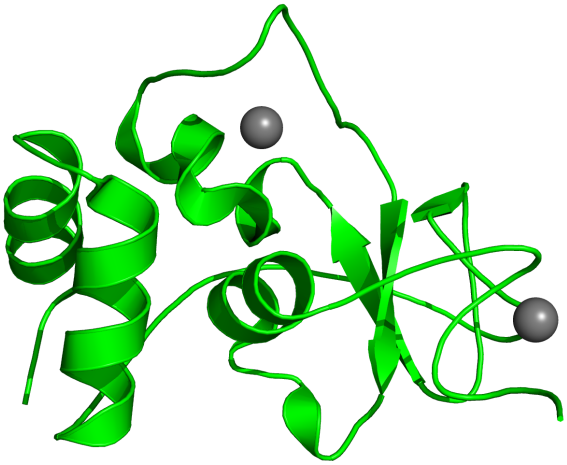

Figure S7 Monomeric modeled structure of Nsp10 protein, with zinc atoms shown as gray spheres.

Uridylate-specific endoribonuclease (Nsp15) and 2'-O-methyltransferase (Nsp16)

The endoribonuclease (Nsp15) and 2'-O-methyltransferase (Nsp16) play vital roles in suppressing the innate immune response. Nsp15 solved experimentally with full amino acid coverage (PDB ID: 6VWW). Nsp16 also has been solved experimentally with full amino acid coverage (PDB ID: 6W4H). Hence no models have been constructed for these proteins.

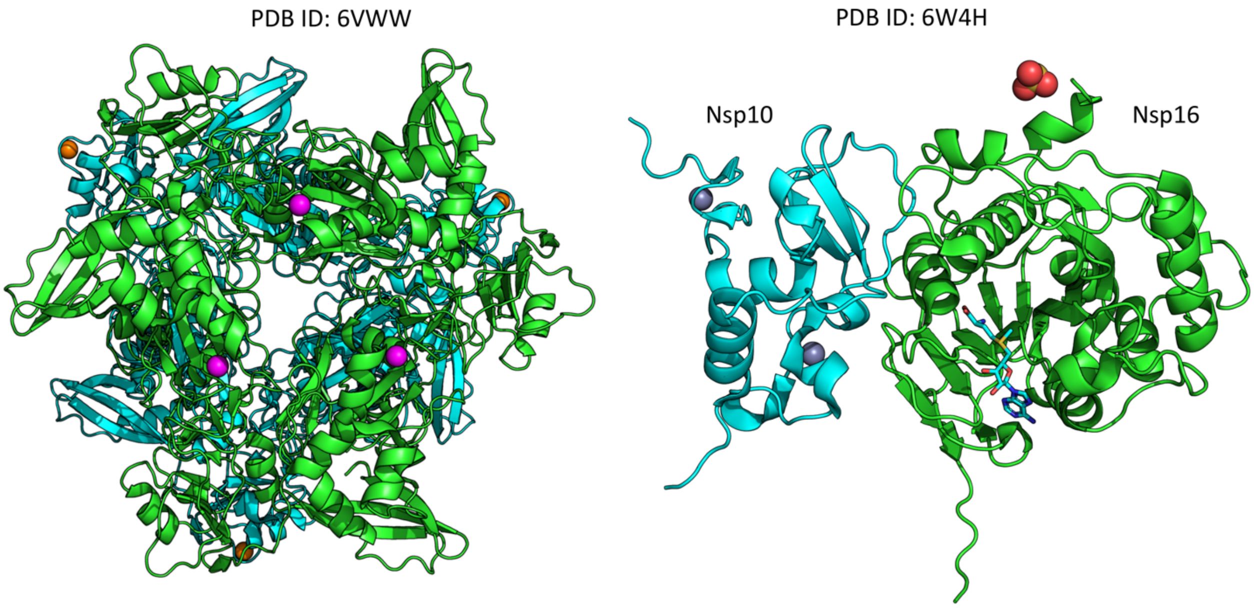

Figure S8 Experimentally solved structure of the Nsp15 (PDB ID: 6VWW) homohexamer, coloured in green and cyan, magnesium ions coloured in magenta, chlorine ions coloured in orange. The experimentally solved structures of the Nsp16-Nsp10 heterodimer (PDB ID: 6W4H) is shown coloured in green and cyan. The S-adenosylmethionine (SAM) ligand is represented in stick cyan. The sulphite ion is shown as a red sphere.

Structural proteins:

Surface glycoprotein (S)

The Spike protein is located on the surface of the virus, available for interaction with human receptors such as ACE2 on order to facilitate virus entry to the human cells. The closed conformation has been modelled using multiple templates (PDB ID: 6M5I, 6YYT, 7C2K) with a MolProbity score of 2.78. while the open conformation has been built with multiple templates (PDB ID: 6CRZ, 2FXP, 3VOP_A, 2LCC_A) with MolProbity of 1.84.

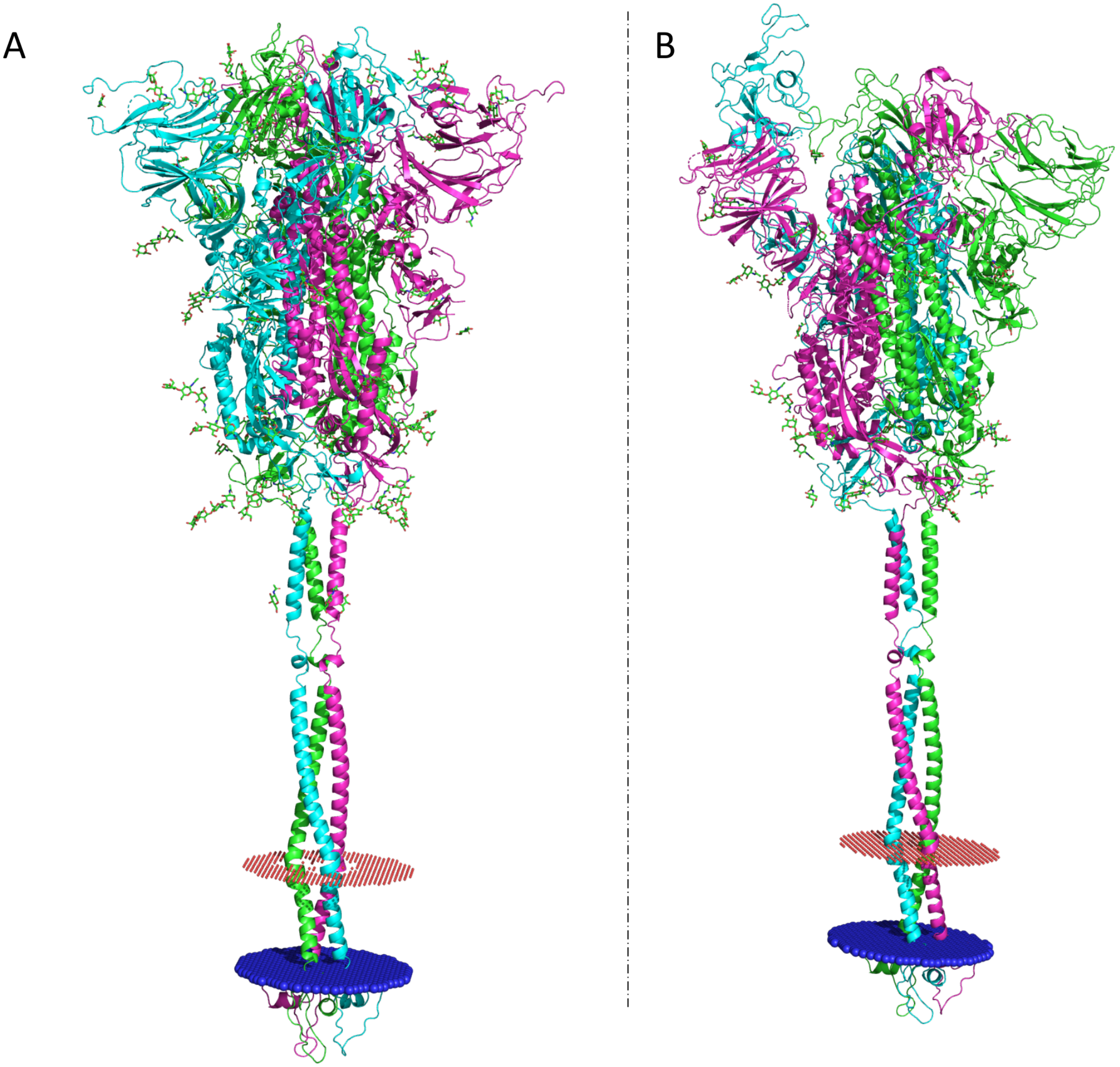

Figure S9 Homotrimer modelled structure of the Spike protein. (A) the closed conformation and (B) the open conformation. Each chain is coloured differently, and the sugar moiety is highlighted in green sticks.

Nucleoprotein (N)

The Nucleoprotein (N) plays a vital role in enhancing viral transcription and replication, It also interact with viral membrane proteins[5]. The process of building this model is described in the Methods section of this manuscript. The modeled structure was built using multiple templates (PDB ID: 6M3M_A, 2CJR_A, 6K12_A, 1F15_A, 5NP3_A) with a MolProbity score of 3.17.

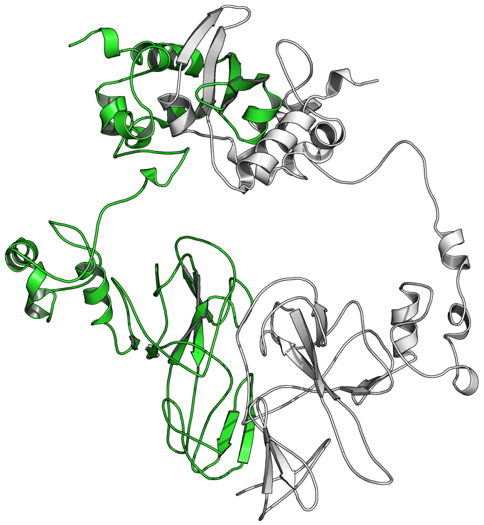

Figure S10 Homodimer model of the Nucleoprotein (N) protein structure, with the equivalent two chains coloured green and grey in order to facilitate visualization of interactions.

Accessory proteins (ORF3a, ORF6, ORF7a, ORF7b, ORF8, ORF10)

ORF3a is the largest accessory protein with 275 amino acids. Its transmembrane protein is located between the Spike and the Envelope proteins. The modeled structure was built using multiple templates (PDB ID: 6XDC, 4BKW_A, 5ZBE_A, 5L2F_A) with a MolProbity score of 2.44. The model of **ORF7a,** another transmembrane protein, was built using multiple templates (PDB ID: 1XAK_A, 1YO4_A, 5XSY_B, 6W37_A) with a MolProbity score of 3. **ORF7b, a** single pass membrane protein, was modeled based on (PDBID: 5XSY_B) with MolProbity of 2.51**. ORF6** suppress host innate immune activation[6]. The modeled structure built using multiple templates (PDB ID: 4GQT_A) with a MolProbity score of 2.37. **ORF8** may play in viral interactions. The modeled structure built using multiple templates (PDB ID: 6P65_A, 5O32_I, 5L74_A) with a MolProbity score of 1.95. **ORF10** is the smallest SARS CoV-2 protein. **ORF9b** play role in inhibition of host innate immune response. the modeled structure built using (PDBID: 6Z4U) with MolProbity score 2.3.

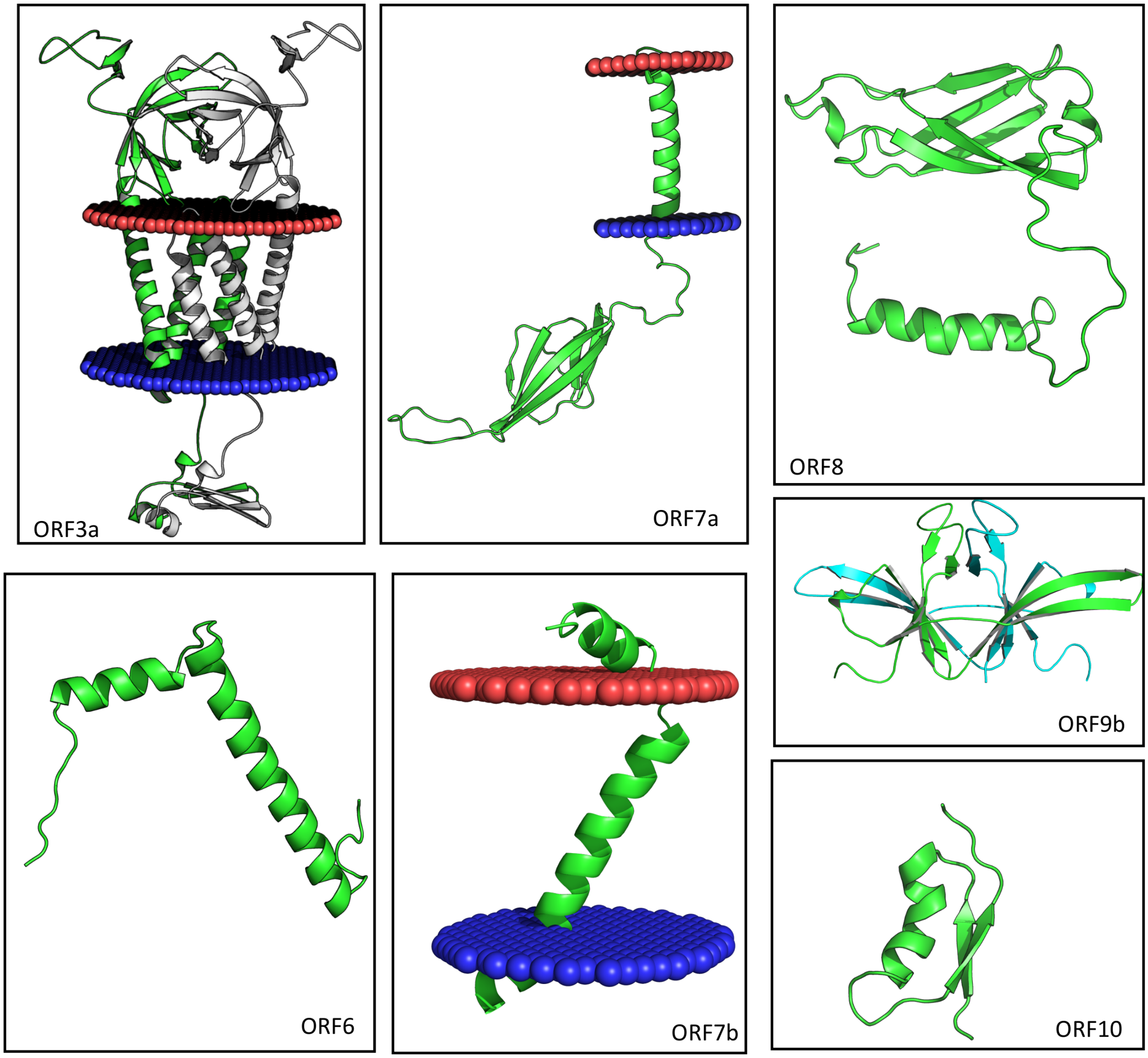

Figure S11 Accessory proteins structure. The transmembrane homodimer modelled structure of ORF 3a protein coloured green and white. Transmembrane monomer modelled structure of ORF7a, ORF7b coloured in green. All the membrane is represented as a red, blue circular structure. ORF6, ORF8, and ORF10 all modeled as monomer and shown in green.

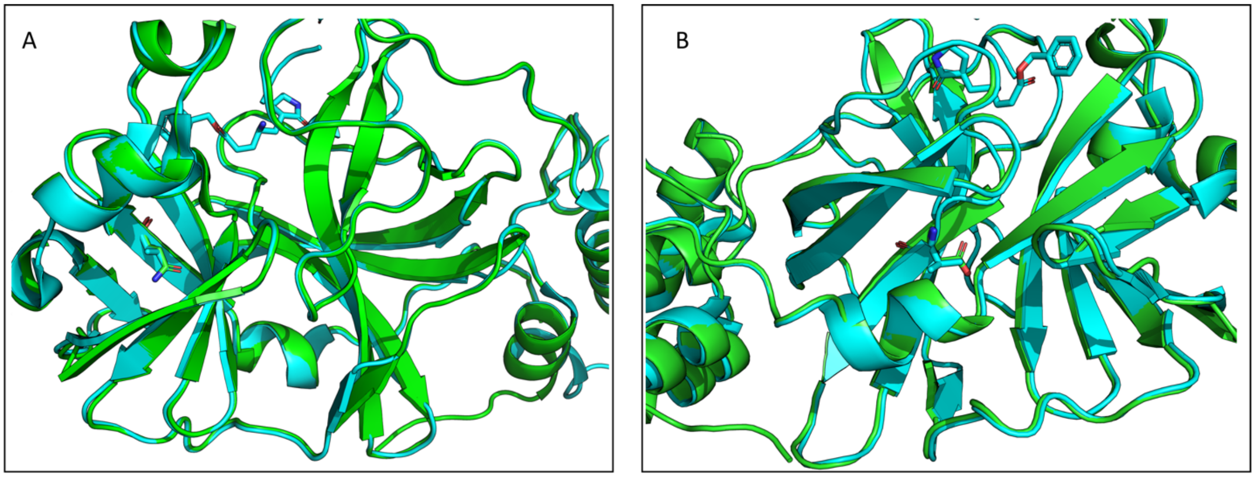

Figure S12 Structural change upon mutations impact for main protease. (A) Nsp5 V20N represented by sticks the wild type structure coloured in cyan, and the mutant structure coloured in green. (B) Nsp5 V148D represented in sticks, the wild type coloured in cyan, whereas the mutant structure coloured in green.

References

1. Ratia K, Saikatendu KS, Santarsiero BD, Barreto N, Baker SC, Stevens RC, et al. Severe acute respiratory syndrome coronavirus papain-like-protease: Structure of a viral deubiquitinating enzyme. Proc Natl Acad Sci U S A. 2006;103:5717–22.
2. Angelini MM, Neuman BW, Buchmeier MJ. Untangling membrane rearrangement in the nidovirales. DNA Cell Biol. 2014;33:122–7.
3. Senior AW, Evans R, Jumper J, Kirkpatrick J, Sifre L, Green T, et al. Improved protein structure prediction using potentials from deep learning. Nature. 2020;577:706–10. doi:10.1038/s41586-019-1923-7.
4. Hillen HS, Kokic G, Farnung L, Dienemann C, Tegunov D, Cramer P. Structure of replicating SARS-CoV-2 polymerase. Nature. 2020;584 August. doi:10.1038/s41586-020-2368-8.
5. Stertz S, Reichelt M, Spiegel M, Kuri T, Martínez-Sobrido L, García-Sastre A, et al. The intracellular sites of early replication and budding of SARS-coronavirus. Virology. 2007;361:304–15.
6. Yuen CK, Lam JY, Wong WM, Mak LF, Wang X, Chu H, et al. SARS-CoV-2 nsp13, nsp14, nsp15 and orf6 function as potent interferon antagonists. Emerg Microbes Infect. 2020;9:1418–28.
